# Optimizing protein yield from growing deer antlers

**DOI:** 10.3389/fbioe.2025.1612239

**Published:** 2025-06-27

**Authors:** Nicolás Alegría-Aravena, María-Pilar López-Garrido, Josefa Quiroz-Troncoso, Raquel González-Martos, Marta Sánchez-Díez, Clara Gavira-O’Neill, Andrés J. García-Díaz, Tomás Landete-Castillejos, Carmen Ramírez-Castillejo, Louis Chonco, Francisco Sánchez-Sánchez

**Affiliations:** ^1^ Instituto de Desarrollo Regional (IDR), Instituto de Recursos Cinegéticos (IREC) and Escuela Técnica Superior de Ingeniéros Agrónomos y Montes y Biotecnología (ETSIAMB), University of Castilla-La Mancha (UCLM), Albacete, Spain; ^2^ Asociación Española Contra el Cáncer (AECC)-Fundación Científica AECC, Albacete, Spain; ^3^ Laboratorio de Genética Médica, Instituto de Biomedicina (IB), Universidad de Castilla-La Mancha (UCLM), Albacete, Spain; ^4^ Centro de Tecnología Biomédica (CTB), Escuela Técnica Superior de Ingeniería Agronómica, Alimentaria y de Biosistemas (ETSIAAB), Universidad Politécnica de Madrid, Madrid, Spain; ^5^ Department of Oncology, Instituto de Investigación Sanitaria San Carlos (IdISSC), Madrid, Spain

**Keywords:** biomedical applications, deer velvet antler, IGF-1, optimization method, protein extraction

## Abstract

Growing antlers in deer contain bioactive compounds, most of which are proteins and peptides with effects on health, such as anticancer and regenerative properties. However, efficient extraction of these biomolecules while preserving their integrity remains a challenge. This study aimed to optimize the extraction of proteins from growing antlers through liquid removal methods, solvent selection, ratios, temperature, and extraction time. Lyophilization was identified as the optimal method for preserving protein integrity, particularly in biologically active regions. Among the tested solvents, water emerged as the most effective for protein extraction, achieving optimal results at a 1:10 w/v ratio with 1 hour of magnetic stirring at room temperature, although remains to be tested the anticancer effect of solvents different to water. Insulin-like growth factor 1 (IGF-1) was quantified as a key indicator of extraction efficiency, demonstrating that the optimized protocol effectively preserves this kind of bioactive protein. This methodology provides a robust framework for the extraction of proteins from growing antlers, paving the way for future applications in biomedical research.

## 1 Introduction

Growing antlers of deer have been used for over 2,000 years in what is called Traditional Chinese Medicine (TCM), a trial-and-error medicine used by most Asian cultures and Russia (Kawtikwar et al., 2010). These bony appendages, which possess the remarkable ability to regenerate annually, are considered one of the fastest-growing tissues in mammals and represent an exceptional biological model for studying tissue regeneration. The fast growth and mineralization is based on protooncogenes, which pose a risk of leading to cancerous growth, exerts an intensive oxidative stress, and the fast mineralization creates such a demand of mineral deposition that induces temporary osteoporosis in the male deer (Wang and Landete-Castillejos, 2023). These highly peculiar characteristics explain some of the proven applications to medicine of the growing antler extract: a wide anticancer activity, anti-aging, tissue regeneration, and promoting mineralization or slowing down bone loss in animal models of osteoporosis (Wu et al., 2013; Cheng et al., 2022; Xia et al., 2022; Wang and Landete-Castillejos, 2023; Rossetti et al., 2024). Some of the biochemical molecules present in antlers, such as proteins, growth factors, amino acids, peptides, and polysaccharides, should be causing one or more of these effects.

Currently, research focuses on properties related to immune system strengthening, tissue regeneration or anticancer potential (Gu et al., 2007; Wu et al., 2013; Li, 2020; Wang and Landete-Castillejos, 2023; Rossetti et al., 2024). However, one of the most important challenges is identifying the compounds responsible for these effects and obtaining them while preserving the integrity of the biomolecules. This process involves considering various factors that can affect the properties and effects of the extracted molecules. These factors include the heterogeneity in the chemical composition of antlers depending on their region (tip, middle, and base), the extraction method employed, and the processing conditions (Gu et al., 2007; Tseng et al., 2014). Additionally, factors such as solvent polarity, temperature, and extraction time have a significant impact on the quantity and quality of the biomolecules obtained (Palma et al., 2013).

In particular, studies have highlighted that growth factors such as IGF-1 (insulin-like growth factor 1) and bioactive peptides present in antlers may be responsible for these beneficial effects. IGF-1 is a key molecule in cellular signaling that regulates cell proliferation and differentiation, and its activity has been linked to regenerative processes and anticancer properties in models both *in vitro* and *in vivo*. Likewise, peptides derived from antlers have demonstrated antioxidant, anti-inflammatory, and anticancer capabilities in various preclinical evaluations, indicating their potential as therapeutic agents (Khan et al., 2025).

According to the literature, the primary candidates for anticancer effects are peptides or proteins, which is why one of the most commonly used solvents for extracting such molecules is water, due to its high polarity (Tang et al., 2019; Zheng et al., 2020; Chonco et al., 2021; Rossetti et al., 2024). The various protocols derived from an extensive literature review reveal numerous factors that can influence the quantity, integrity, and functional characteristics of the biomolecules. These include water removal processes, solvent polarity, and temperatures, not to mention all the inherent factors related to the specific characteristics of the specimen being studied (Ren et al., 2019; Yao et al., 2019). To date, the main active effectors derived from DVA (deer velvet antlers) have been described as proteins ranging from 0.5 to 2,000 kDa (Sun et al., 2023). These proteins can perform various beneficial functions for human health, as they are involved in chronic and degenerative diseases. For instance, a recent approach evaluated the efficiency of antler stem cell-derived exosomes, demonstrating that these nanoscale vehicles contain key proteins that can modulate the tumor microenvironment and enhance the efficacy of immunotherapies, such as immune checkpoint inhibitors (Zhang et al., 2023a; 2023b; Zhou et al., 2024).

Therefore, it is essential to extract as many of these molecules as possible to maximize their properties, not only to advance the understanding of their biochemical composition but also to establish their potential in biomedical applications. The preservation of biomolecules such as IGF-1 and other growth factors during the extraction process represents a top priority, as their integrity is crucial to maintaining their biological functions.

In this study, we present a methodological approach to extract proteins from growing antlers efficiently. To achieve this, a solvent gradient and variations in extraction time were employed. Subsequently, under optimal conditions, IGF-1 concentration was used as an indicator of the protocol efficiency in preserving and extracting bioactive proteins of interest, with potential implications for biomedical applications.

## 2 Methods

### 2.1 Materials and equipment

All equipment used is shown in Table 1. Reagents are shown in Table 2. Solvent characteristics are shown in Table 3.

**TABLE 1:** Equipment used in the research.

Equipment	Model	Supplier	Country
Lyophilizer	BIOBASE BK-FD10PT	BIOBASE BIODUSTY	Wolfenbüttel, Germany
Hot-drier	DIGITHEAT 80L	TQTECH	Barcelona, Spain
Sublimator	30EKS	ZIRBUS Technology GmbH	Bad Grund, Germany
Blade mill	Retsch SM300	Retsch GmbH	Haan, Germany
Mixer mill	Retsch MM400	Retsch GmbH	Haan, Germany
Magnetic stirrer	SBS-MR-1600/6	Steinberg Media Technologies GmbH	Hamburg, Germany
Homogenizer	POLYTRON PT 2100	Kinematica AG	Lucerne, Switzerland
Microplate reader	BIOBASE-EL 10th	BIOBASE BIODUSTY	Wolfenbüttel, Germany
Microplate reader	Epoch	Biotech	Vermont, United States
Vortex	ZX3	VELP Scientifica	Bohemia, New York, United States
Miliq Dispenser	Ultramatic GR	Wasserlab	Navarra, Spain
Microplate shaker	PMS-1000i	GRANT bio	Cambridge, United Kingdom
GraphPad prism	8.0.1	GraphPad Software Inc.	San Diego, CA, United States

**TABLE 2:** Reagents used in the research.

Reagents	Code	Supplier	Country
Ethanol (EtOH)	ETHA-9TP-1K0	Labbox	Barcelona, Spain
Acetone (Acet)	ACET-0IA-1K0	Labbox	Barcelona, Spain
Acetonitrile (ACN)	ACTN-0GH-2K5	Labbox	Barcelona, Spain
BCA Protein Assay Kit	71285-M	MerckMillipore	Massachusetts, United States
Coomasie blue G-250 5 mg	1.15444	Sigma Aldrich	Burlington, Massachusetts, United States
Orthophosphoric acid	345245	Sigma Aldrich	Burlington, Massachusetts, United States
Phosphate-Buffered Saline (PBS) 10X	SH30258.01	Cytiva	Burlington, Massachusetts, United States
ELISA kit	CSB-E12644De	Cusabio	Wuhan, China

**TABLE 3:** Solvents characteristics.

Solvent	Polarity index	Proticity	Denaturing level	Dielectric constant	Viscosity
Water	10.2	Protic	Low	∼80	0.89 mPa s
Ethanol	5.2	Protic	Moderate	∼24.3	1.2 mPa s
70° ethanol	7.2	Protic	Moderate	∼55	∼1.3 mPa s
40° ethanol	8.2	Protic	Moderate	∼65–70	∼1.5 mPa s
Acetone	5.1	Aprotic	High	20.7	0.32 mPa s
70° acetone	7	Aprotic	High	∼55	∼0.6 mPa s
40° acetone	8	Aprotic	High	∼65–70	∼0.8 mPa s
Acetonitrile	5.8	Aprotic	Low	37.5	0.34 mPa s
70°Acetonitrile	7	Aprotic	Low	∼55	∼0.5 mPa s
40°Acetonitrile	8	Aprotic	Low	∼65–70	∼0.6 mPa s

### 2.2 Methods

**FIGURE 1:**
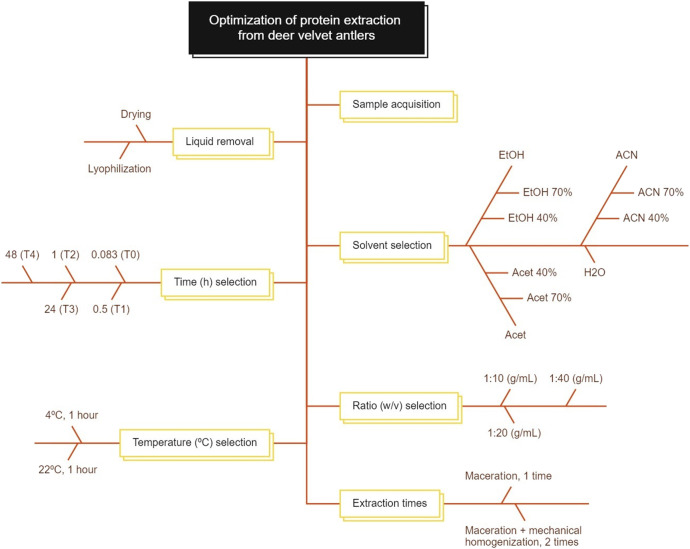
Procedures to optimize protein extraction from growing antlers of deer. The methodology is employed to identify an optimized protocol for protein extraction efficiency. This involves a series of steps, beginning with the removal of liquid, the selection of an appropriate solvent, the determination of the optimal extraction time, the calculation of the solvent-to-sample ratio, the identification of an appropriate extraction temperature, the estimation of the number of protein extraction repetitions, and finally, the determination of the time/acceleration ratio for the final centrifugation.

The procedure optimized in this paper has been scheduled in Figure 1, and explained in the following sections:

#### 2.2.1 Material acquisition

Four antlers of red deer were collected from animals shot for other purposes (selective hunting to reduce population) in the province of Ciudad Real (Castilla-La Mancha, Spain) and stored at −80°C. The antlers were donated by the private game estate “La Morera,” located in the municipality of Abenójar, Ciudad Real, Spain, following the culling of the deer by population control. The slaughter of the hunted animals was regulated by the Regional Hunting Law of Castilla la Mancha (Law 3/2015, dated 5th of March, of Hunting in Castilla-La Mancha. Published in Diario Oficial de Castilla-La Mancha 49, 7,039–7,097 (2015) modified by Law 2/2018, dated 15th of March, that modifies the mentioned law 3/2015. Published, in turn, in Diario Oficial de Castilla-La Mancha 60, 8,888–8,916 (2018)).

The antlers were in a size and shape compatible with antlers at the 60-day growth stage after casting (the harvesting stage for TCM). This is based on the expertise in raising experimental deer from UCLM and the guidelines of Deer Industry New Zealand for antler harvesting.

#### 2.2.2 Comparison of hot-drying vs. lyophilization of samples

**FIGURE 2:**
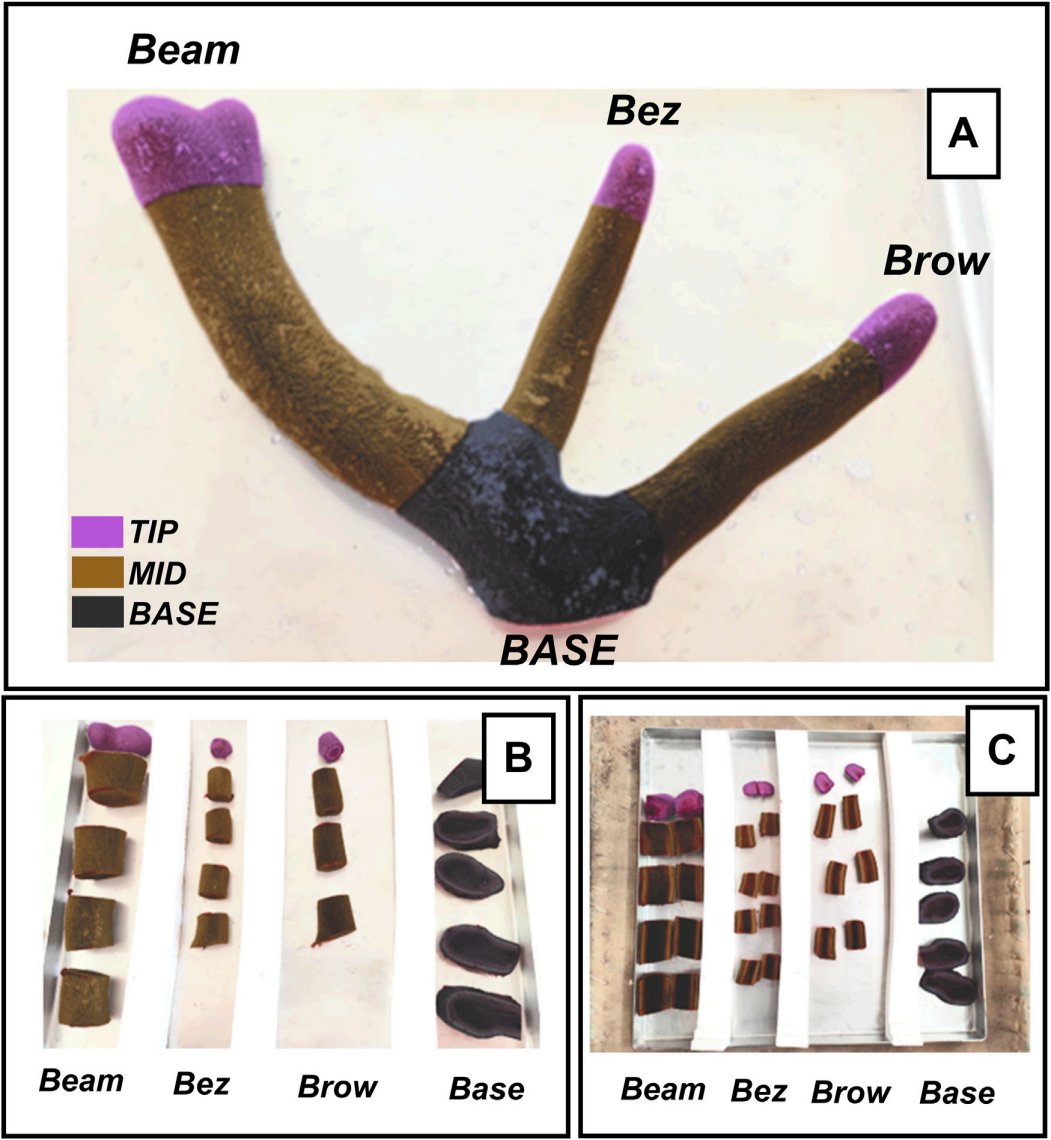
Antler before removal of liquids. **(A)** Uncut antler. **(B)** Antler cut into four different segments by sections of the antler (base, brow, bez and beam). **(C)** Dried antler, longitudinally cut sections represented how TIP (violet, 2.5 cm), MID (brown, middle zones of 5 cm), and BASE (rest of the antler, black).

In order to ascertain the optimal methodology for the removal of water from the antler sample, a comparison was conducted between the hot-drying method and lyophilization. Figure 2 represents an example of the antlers divided into different segments, with each segment corresponding to a specific branch. The segments were labelled as follows: base, brow tine, bez tine and beam (Figure 2). The first branch is the brow tine (right in Figure 2A), located slightly higher than the base and is usually more curved and closer to the base. The bez tine is the second segment (middle in Figure 2A). The final branch is the main beam and curves outward from the top of the antler. The sections were made with 5 cm cuts, except for the tip, which was 2.5 cm (violet in Figures 2A–C).

Antler sections were hot-dried (in a stove brand TQTECH DIGITHEAT 80L, Barcelona, Spain at 60°C for 14 days), while the remaining sections were lyophilised in a Sublimator 30 EKS (ZIRBUS technology GmbH, Bad Grund, Germany) at 0.2 mbar for 46 h (condenser temperature, −45°C). The prepared samples were then ground using a blade mill at 1,500 rpm and 2 mm pore size (Retsch SM300, Retsch GmbH, Haan, Germany) and then mixed with a mixer mill at 30 Hz for 2 min until a grain size of 0.18 mm was achieved (Retsch MM400, Retsch GmbH, Haan, Germany).

For the optimization, Tip from Beam antlers were used in each step, using three to five different deer for the assays.

#### 2.2.3 Solvent and time optimization

After selecting the best method for water removal from the antler sample while maintaining the integrity of the molecules, the solvent capable of extracting the highest amount of proteins was determined. For this, the ground bottom antler was homogenized in magnetic stirrer with heating plate SBS-MR-1600/6 (Steinberg Media Technologies GmbH, Hamburg, Germany) at a ratio of 1:10 (grams/mL) with different solvents: Milli-Q grade water (H_2_O), 100% ethanol (EtOH), 70% ethanol (EtOH 70%), 40% ethanol (EtOH 40%), 100% acetone (Acet), 70% acetone (Acet 70%), 40% acetone (Acet 40%), 100% acetonitrile (ACN), 70% acetonitrile (ACN 70%), and 40% acetonitrile (ACN 40%). Each condition with different times corresponding to 5 s in contact to vortex (T0), 30 min (T1), 1 h (T2), 24 h (T3), and 48 h (T4) at room temperature and 400 rpm with microplate shaker PMS-1000i (GRANT bio, Cambridge, United Kingdom), resulting in 50 experimental conditions (n = 4).

#### 2.2.4 Optimization of extraction ratio and temperature

Once the best protein extraction method was determined, with the aim of optimizing the best grinding ratio and solvent, the ground growing deer antler was homogenized at ratios of 1:10, 1:20, and 1:40 (grams/mL). Each condition is homogenized at room temperature. Finally, it were compare at room temperature and 4°C for 1 h of extraction, using the best grinding ratio condition.

#### 2.2.5 Double extraction

Once the sample processing parameters and the first extraction were determined regarding solvent, ratio (sample), and temperature, a test was conducted to see if performing a second extraction increases the total protein content. For this, after completing the first extraction by maceration, a mechanical homogenization was performed at 500 W, 20,000 rpm with a speed of 22 m/s for 2 min (POLYTRON PT 2100, Kinematica AG, Lucerne, Switzerland).

#### 2.2.6 Protein quantification

Protein concentration was determined by BCA Protein Assay Kit (MerckMillipore, Massachusetts, United States) or by Bradford method (coomasie blue G-250 5 mg, ethanol 2.5 mL, orthophosphoric acid 5 mL, make up to 50 mL with distilled water).

For BCA detection, a BSA standard line was used with the points of 2, 1, 0.5, 0.25, 0.125, 0.0625, 0.03125, 0.015625 and 0 mg/mL diluted with Milli-Q water. The samples were diluted at a ratio of 1:50 in order to fall within the interpolable range of the reference curve with Milli-Q water. The reaction was then measured at 562 nm using a microplate reader (BIOBASE-EL 10th, BIOBASE BIODUSTY, Wolfenbüttel, Germany).

For Bradford detection, a BSA standard line was used with the points 0.3, 0.2, 0.1, 0.05, and 0 mg/mL. Samples were diluted at a ratio of 1:200 to fall within the interpolable range of the reference curve. The reaction was measured at 595 nm using a microplate reader Epoch (Biotech, Vermont, United States).

#### 2.2.7 Deer IGF protein detection and quantification

With the best parameters obtained from the standardization and optimization of the method, ELISA was performed for IGF-1. The detection and quantification of deer insulin-like growth factor (IGF-1) was carried out from 1 mL of sample resuspended in PBS 1X after lyophilisation and using the ELISA method with a kit (REF: CSB-E12644De; Cusabio, Wuhan, China) following the manufacturer’s recommendations, except for the final incubation step of the protocol, which was performed for 6–8 min instead of the recommended 15 min to avoid colour saturation.

#### 2.2.8 Statistical analysis

GraphPad Prism 8.0.1 (GraphPad Software Inc., San Diego, CA, United States) was used for statistical analysis with one-way parametric analysis of variance (ANOVA) to compare normally distributed groups and non-parametric analysis for outliers (Figure 4). Student’s t-test were applied in other figures. The significant differences are indicated as *** for p < 0.001, ** for p < 0.01, and * for p < 0.05.

## 3 Results

### 3.1 Drying and lyophilization of deer antler

**FIGURE 3:**
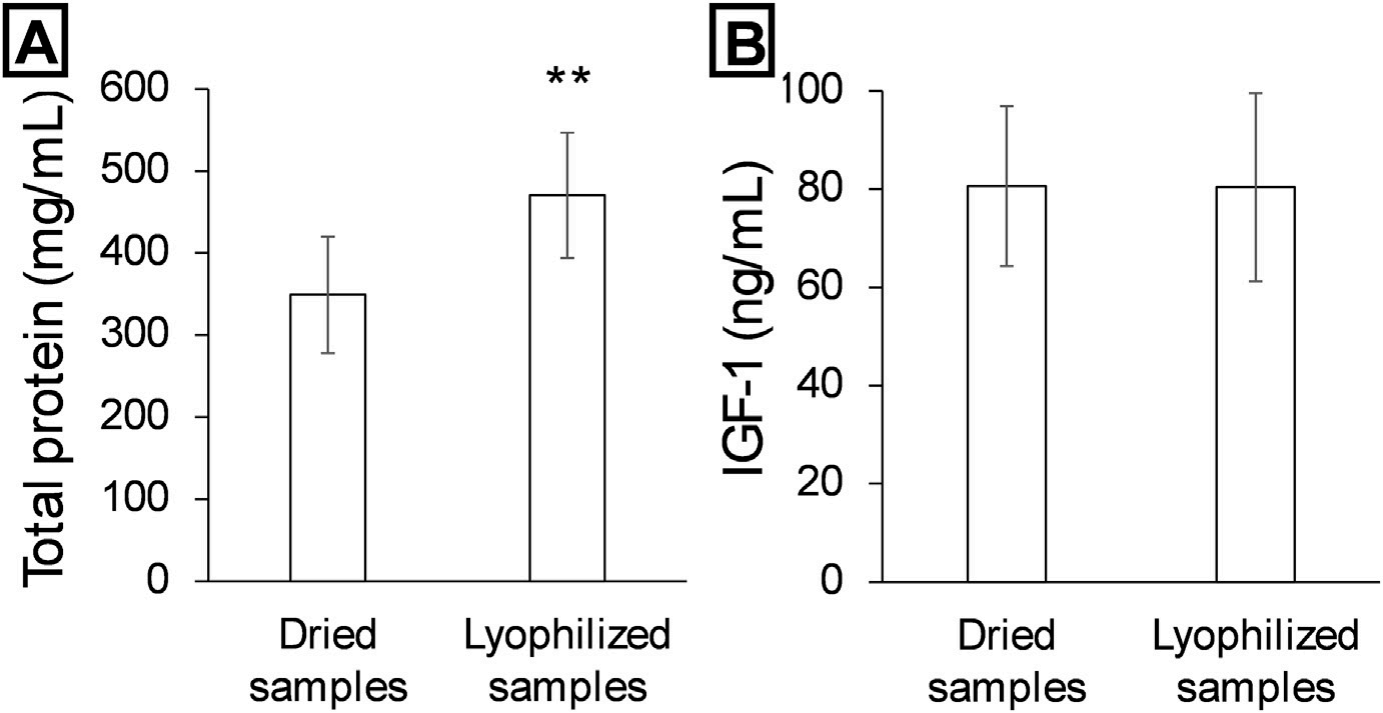
Liquid removal methods comparison from deer antler velvet. Detection of total proteins **(A)** and IGF-1 **(B)** using a first desiccation or freeze-drying step from the deer antler section. Data are presented as mean ± SEM (n = 3). **P < 0.01.

Two treatments were compared to determine the best method for preserving the sample before pulverization: heat-drying and lyophilization. Total protein quantification revealed a statistically significant difference between the two methods (Figure 3A), with a higher protein concentration in the lyophilized samples (approximately 500 mg/mL) compared to the heat-dried samples (approximately 350 mg/mL) (p < 0.01).

In contrast, IGF-1 levels did not differ significantly between the two treatments (Figure 3B), remaining close to 85–90 ng/mL in both conditions.

### 3.2 Optimization of both type of solvent and extraction time

**FIGURE 4:**
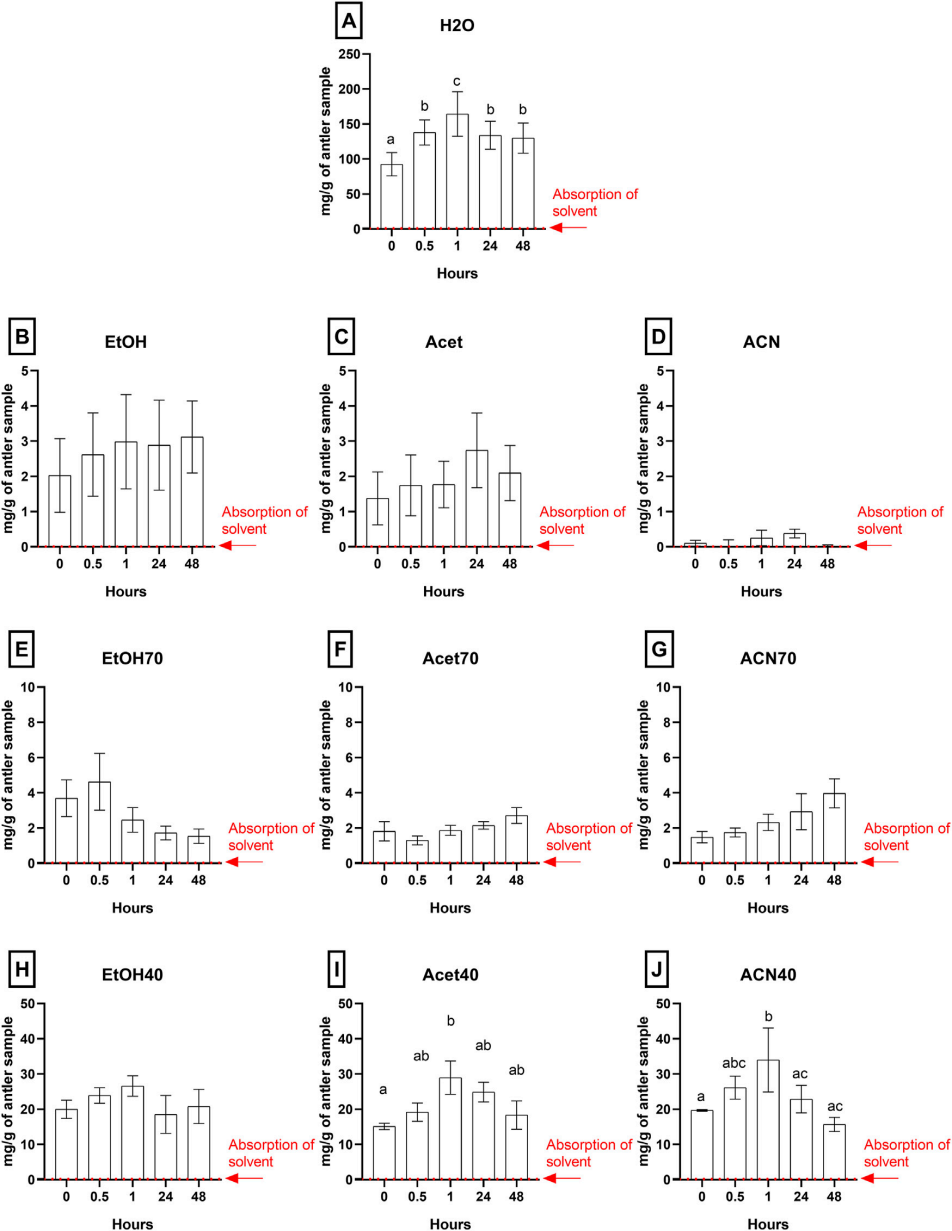
Solvent and time gradient extraction. Protein extraction using different solvents: water **(A)**, ethanol **(B)**, acetone **(C)**, acetonitrile **(D)**, 70% ethanol **(E)**, 70% acetone **(F)**, 70% acetonitrile **(G)**, 40% ethanol **(H)**, 40% acetone **(I)**, and 40% acetonitrile **(J)**, at various time intervals: zero time (considering how vortex contact), 30 min, 1 h, 24 h, and 48 h. Data are presented as mean ± SEM (n = 4). Bars or data points not sharing the same letter are significantly different (p < 0.05) between sterilization methods. significant differences are indicated as *** for p < 0.001, ** for p < 0.01, and * for p < 0.05.

The efficiency of ten protein extraction solvents (water, ethanol, acetone, acetonitrile, and their combinations at 40% and 70%) was evaluated under magnetic stirring at three different times (0.5, 1, and 4 h). As shown in Figure 4, the highest protein yield was obtained with distilled water after 1 h of extraction (Figure 4A), reaching significantly higher concentrations compared to the other solvents and times analyzed (p < 0.001).

The extracts obtained with ethanol (Figures 4B, E), acetone (Figures 4C, F), and acetonitrile (Figures 4D, G) showed no significant differences between the different times, remaining relatively constant but at low levels. In particular, the solvents with the highest concentration of acetonitrile, acetone, and pure ethanol, showed limited efficiency for protein extraction.

In contrast, the extracts obtained with a 40% (Figures 4I, J), although they exhibited a progressive increase over time, did not reach the yield observed with water. On other hand, Figure 4H shows a constant protein concentration over time. This is mainly because longer incubation times do not enhance extraction and instead promote protein degradation. The results are shown in Table 4.

**TABLE 4:** Optimal extraction in relation solvent/time.

Solvent	Optimal time (h)	Max extraction (mg/g)	Error (±mg/g)
H_2_O	1	164.3	31.8
Et OH	1	3.0	1.3
Acet	24	2.7	1.1
ACN	24	0.4	0.1
EtOH 70%	0.5	4.6	1.6
Acet 70%	48	2.7	0.4
ACN 70%	48	4.0	0.8
EtOH 40%	1	26.6	2.9
Acet 40%	1	29.0	4.7
ACN 40%	1	34.0	9.1

### 3.3 Ratio and temperature optimization

**FIGURE 5:**
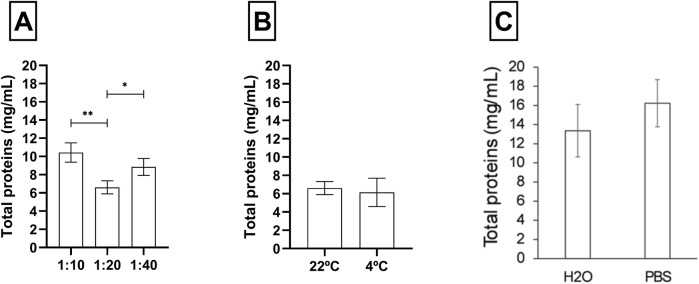
Ratio, temperature, and water/PBS extraction comparison. **(A)** Ratio comparison between 1:10, 1:20, and 1:40 weight: volume. **(B)** Total proteins of temperature comparison between room temperature and 4°C. **(C)** Detection of total proteins using H_2_O and PBS (0.1 M) as solvent of choice. Data are presented as mean ± SEM (n = 3). The significant differences are indicated as **p < 0.01, *p < 0.05.

Protein extraction efficiency was evaluated using two aqueous solvents: distilled water (H_2_O) and phosphate-buffered saline (PBS 1X) at different extraction ratios. As shown in Figure 5, the use of PBS produced a slightly higher protein concentration (∼16 mg/mL) compared to water (∼13 mg/mL). However, this difference was not statistically significant, as the standard deviations overlapped between the two groups. Along with these data, it was determined that a 1:10 ratio offered the best balance between yield and manageability, as lower ratios produced an overly dense mixture, while higher ratios excessively diluted the sample.

Finally, a comparison between extraction temperature conditions: 4°C and room temperature showed no significant differences in extraction yield. Therefore, a 1-h incubation at room temperature was selected as the standard condition.

### 3.4 Single vs. double extraction

To evaluate the impact of a second mechanical extraction, the results of a single extraction performed with or without a mechanical homogenizer were compared.

**FIGURE 6:**
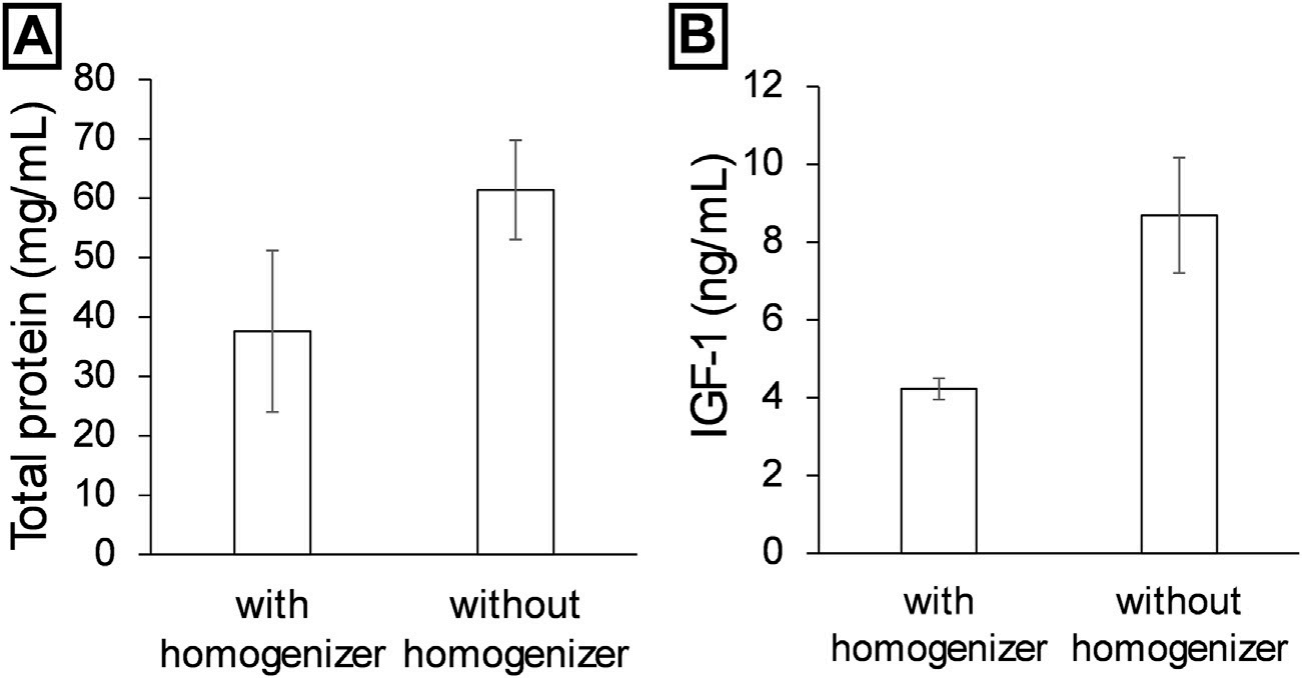
Comparison of protein extraction using or not using a homogenizer. Comparison of double extraction using maceration and a homogenizer (polytron) with using a single maceration step. **(A)** is comparison respect at total protein, and **(B)** is the comparison of IGF-1. Data are presented as mean ± SEM (n = 2).

Figure 6A shows that samples extracted without homogenization had significantly higher total protein concentrations, with values reaching approximately 60 mg/mL, compared to samples treated with a homogenizer, which recorded concentrations close to 35 mg/mL. Figure 6B shows a similar pattern for IGF-1, whose concentration was also higher in samples extracted without homogenization, practically doubling the levels obtained with homogenization (∼9 ng/mL vs. ∼4 ng/mL, respectively). Applying Cohen’s d, a value of approximately 4.91 was obtained for total protein measurement, with a statistical power of around 70.7%. In contrast, for IGF-1 quantification, Cohen’s d was approximately 2.84, with a statistical power of 35.8%. Given the large observed differences, the data from the graphs suggest biologically significant differences.

### 3.5 Concentration of the sample

Finally, to improve the detectability of bioactive proteins in the deer antler extract, the impact of supernatant concentration and lyophilization was evaluated. After removing colloidal matter by centrifugation, adjusting speed and time to optimize sedimentation, lyophilized and non-lyophilized extracts were compared.

**FIGURE 7:**
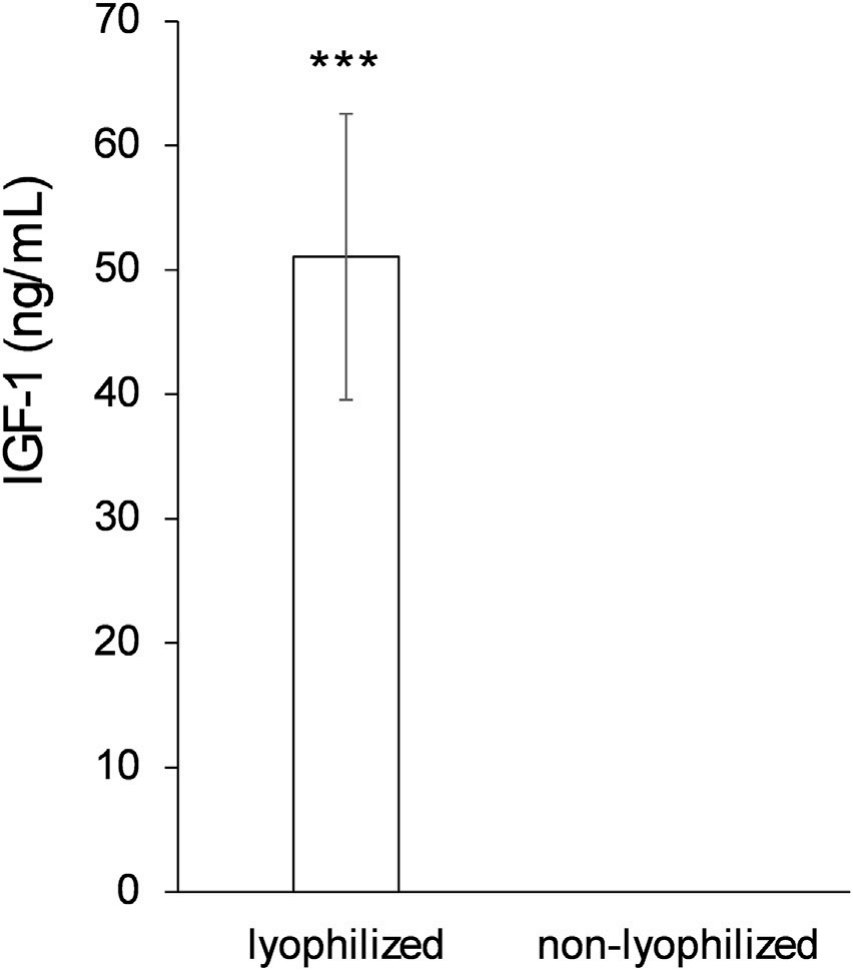
Final lyophilization process comparison. IGF-1 detection after protein extraction using a final freeze-drying step or not to concentrate the sample. Data are presented as mean ± SEM (n = 5). *** P < 0.001.

As shown in Figure 7, ELISA quantification revealed that IGF-1 was detectable only in the lyophilized extract, with a concentration above 50 ng/mL. In contrast, IGF-1 levels were undetectable in the non-lyophilized extract. This difference was highly significant (p < 0.001).

## 4 Discussion

The goal in obtaining these molecules is not only to maximize their extraction but also to ensure that they retain their activities for future application and use. Lyophilisation preserves the integrity of the tissue, particularly the cartilaginous and vascular regions (Mahirogullari et al., 2007; Li et al., 2024). These areas are highlighted because they correspond to zones of high biological activity for growth factors and structural proteins (Li et al., 2005; Ba et al., 2019). Various studies explain that, after hydrating proteins, water removal thermodynamically affects protein folding (Matejtschuk, 2007; Kasper and Friess, 2011; Molnar et al., 2021). Therefore, lyophilization emerges as the best method for water removal compared to conventional dehydration, as it eliminates water without subjecting proteins to high temperatures or harsh conditions that could denature them. This process preserves the three-dimensional structure and functionality of many biomolecules; however, not all proteins are equally susceptible. For instance, insulin-like growth factor 1 (IGF-1) is less labile and can better tolerate certain water removal processes without undergoing significant alterations in its structure or function (Ortega Castillo et al., 2009; Karunnanithy et al., 2024). This factor was used as a reference for all assays, as it is mainly present during the antler growth stage, playing a role in cell proliferation, tissue regeneration, and antler ossification. IGF-1 is present at low concentrations and yet its signal can still be detected, which validates, improves the analytical sensitivity, and ensures the quality of the extraction protocol.

The wide range of molecules present in antlers have different effects on human health. Extensive reviews show the impact of extracts obtained through enzymes, fermentation, organic solvents, and aqueous solvents (Wu et al., 2013; Xia et al., 2022). TCM use of growing antlers of deer is mostly used with water extraction (Kawtikwar et al., 2010), although alcohol one is also used. Other studies have described that these extracts obtained with aqueous solvents exhibit anti-osteoporosis activity (Zhang et al., 2013; Tseng et al., 2016; REN et al., 2019), anti-arthritis activity (Kim et al., 2004; 2005; Cheng et al., 2022), anti-inflammatory activity (Zhao et al., 2016; Kuo et al., 2018; Yao et al., 2018; Cheng et al., 2022), immunomodulatory activity (Yao et al., 2018), antioxidant activity (Li et al., 2020), antidopaminergic activity (Kim and Lim, 1999), neuroprotective activity (Kim et al., 1999; 2014; Liu et al., 2024), and anticancer activity (Tang et al., 2019; Zheng et al., 2020; Chonco et al., 2021; Rossetti et al., 2024). However, the various components extracted with other solvents such as ethanol, acetone, or acetic acid also possess several properties, among which antioxidant capacity stands out (Xia et al., 2022), but also anticancer ones (Yang et al., 2017; Yao et al., 2018). The differences between extracts are due to the characteristics of the solvents. Table 1 adequately explains the low protein extraction yield of ethanol and acetone. Both solvents have high levels of protein denaturation due to dehydration. As their concentration decreases, they are better able to capture proteins. On the other hand, acetonitrile has a low denaturing effect; however, it lacks the ability to donate hydrogen for bond formation and molecule capture.

Water was selected as the best solvent, not only because it extracts the majority of the protein population and has shown positive results in previous studies, but also because the clinical applications or biomedical applications of ethanol, acetone, and acetonitrile have not been detailed in literature.

Although PBS was more efficient in protein extraction than water (without significant differences; Figure 5), it was decided to standardise the extraction with water to avoid the concentration of salts in a subsequent lyophilisation step. The main difference we can find between extraction with water and a buffer is that a low salt concentration increases the solubility of proteins. Conversely, if the salt concentration is high, the proteins will precipitate (Hassan, 2005). If we consider a scaling of the process, water solvent allows a greater productivity at industrial level to generate extracts without needing a final step of dialysis or concentration of protein. Authors have shown that a 1:10 ratio maintains adequate mass transfer and dissolution without promoting thermal degradation in processes involving heat and optimizes the efficiency of compound extraction (Wang et al., 2018; Zhang et al., 2018; Crăciun and Gutt, 2023; Popova and Bankova, 2023). On the other hand, after testing two temperatures at different time intervals, no significant differences were detected when comparing extraction at room temperature and at 4°C. Considering that homogenization at room temperature improves the solubility of molecules and increases the extraction rate, and that extraction at 4°C preserves temperature-sensitive molecules, both approaches have their respective advantages (Chemat et al., 2020; Huber et al., 2021; Shi et al., 2022).

In other hand, a second extraction was performed to improve extraction efficiency. According to several studies, repeating the process or using a second solvent has shown significant improvements in the final yield (Luo et al., 2017; Villaret-Cazadamont et al., 2020). In general, double extraction allows for maximizing compound recovery and increases the purity of the extract if the solvents used are changed (Martínez-Maqueda et al., 2013; Yang et al., 2021). Without homogenization, an increase in the amount of total protein is observed (up to 66% more), and twice the amount of IGF-1 is extracted. However, this does not mean that these data are absolute, as cellular content, favoured by the use of the homogenizer, may cause interference in detections, which would confirm the increase in extract purity for certain types of proteins.

Finally, the results obtained demonstrate the need for a sample concentration step for the correct detection of biomolecules in the extract. Although the centrifugation process eliminated solid impurities and colloidal components, some soluble molecules, especially those present at low concentrations, remain below the detection limit if a concentration procedure is not applied.

Lyophilization proved to be an effective strategy for increasing the detection sensitivity of specific proteins such as IGF-1. This result is consistent with previous studies indicating that many growth factors or bioactive peptides are present in trace amounts in complex biological matrices and require prior concentration for quantitative analysis using immunoenzymatic techniques such as ELISA.

In addition to its ability to concentrate the analyte, lyophilization offers the advantage of preserving the structural stability of heat-sensitive proteins, maintaining their biological activity intact. Therefore, this final procedure was incorporated as a standard step in the preparation of the protein extract, ensuring the recovery and detection of functionally relevant components.

All these results do not mean that water is the only solvent able to extract biomolecules with biomedicine applications (i.e., there may be other molecules with medical applications not soluble in water). However, water is the solvent with the greatest capacity to extract protein (and most molecules with medical applications are likely to be proteins). It is important to assess if extracts with solvents different to water also have biomedical application, even if water is already showing many of these.

**FIGURE 8:**
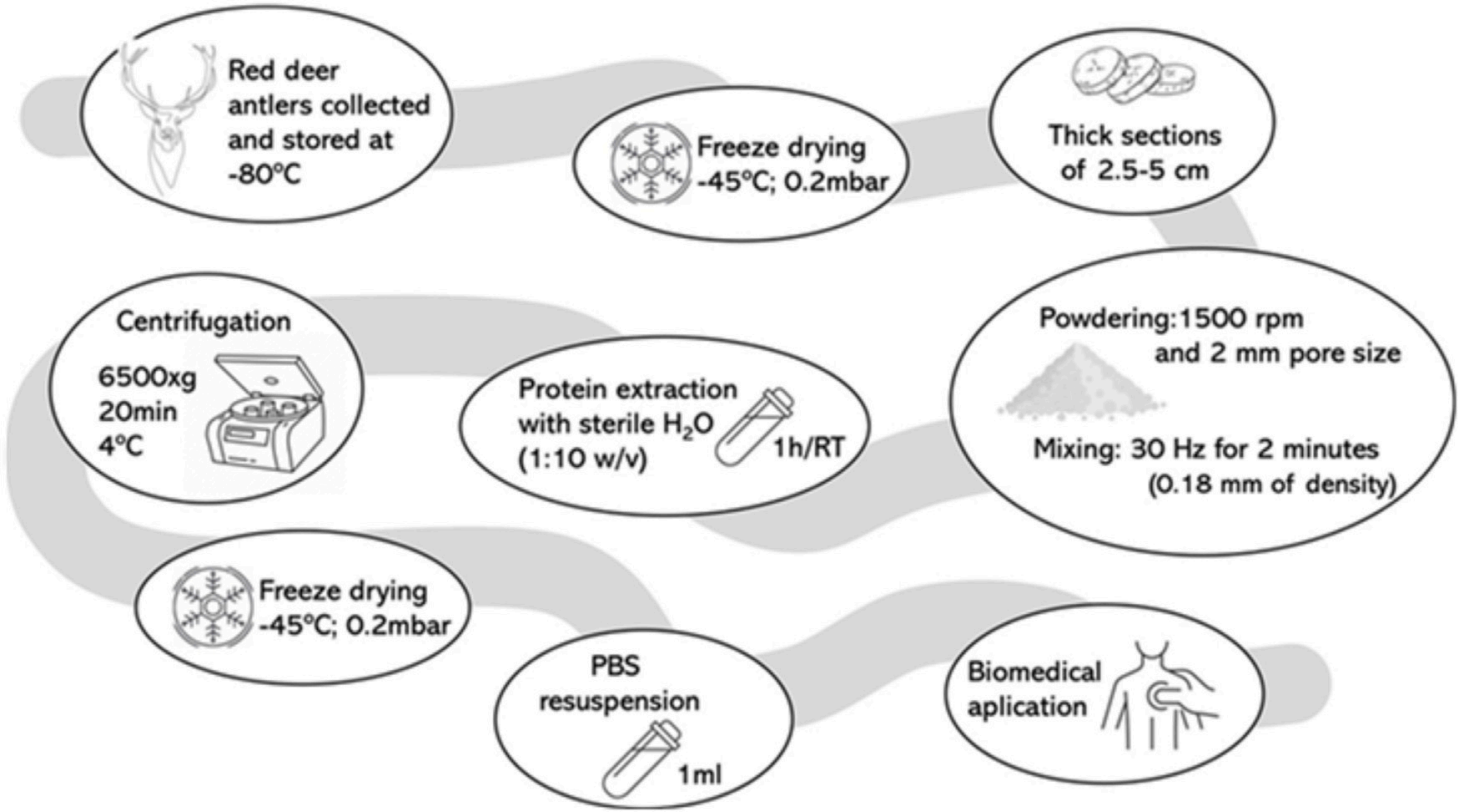
Final protocol for the protein extraction for deer velvet antler. RT, room temperature.

In conclusion, the optimal conditions for extracting bioactive compounds from growing antlers, focusing primarily on protein content, involve obtaining the antlers, freezing them at −80°C for lyophilisation, and cutting them into 2.5–5 cm thick sections. Subsequently, milling the material and performing molecular extraction in a 1:10 w/v ratio with sterile Milli-Q water for 1 h at room temperature. Finally, the optimal method involves centrifuge at 6,500 × g for 20 min at 4°C, and lyophilize the supernatant to concentrate the biomolecules, resuspending them in 1X PBS (NaCl 27 g/L; KCl 0.2 g/L; Na2HPO4 1.15 g/L; KH2PO4 0.2 g/L, adjust pH to 7.4). Figure 8 represents the final protocol for protein extraction for DVA.

## Data Availability

The original contributions presented in the study are included in the article/Supplementary Material, further inquiries can be directed to the corresponding authors.

## References

[B1] Ba H. Wang D. Yau T. O. Shang Y. Li C. (2019). Transcriptomic analysis of different tissue layers in antler growth Center in Sika Deer (Cervus nippon). BMC Genomics 20, 173–13. 10.1186/S12864-019-5560-1 30836939 PMC6402185

[B2] Chemat F. Abert Vian M. Fabiano-Tixier A. S. Nutrizio M. Režek Jambrak A. Munekata P. E. S. (2020). A review of sustainable and intensified techniques for extraction of food and natural products. Green Chem. 22, 2325–2353. 10.1039/C9GC03878G

[B3] Cheng W. J. Yang H. T. Chiang C. C. Lai K. H. Chen Y. L. Shih H. L. (2022). Deer velvet antler extracts exert anti-inflammatory and anti-arthritic effects on human rheumatoid arthritis fibroblast-like synoviocytes and distinct mouse arthritis. Am. J. Chin. Med. (Gard City N Y) 50, 1617–1643. 10.1142/S0192415X22500689 35850642

[B4] Chonco L. Landete-Castillejos T. Serrano-Heras G. Serrano M. P. Pérez-Barbería F. J. González-Armesto C. (2021). Anti-tumour activity of deer growing antlers and its potential applications in the treatment of malignant gliomas. Sci. Rep. 11, 42. 10.1038/S41598-020-79779-W 33420194 PMC7794318

[B5] Crăciun A. L. Gutt G. (2023). Optimization of experimental parameters in the solvent extraction of trans-resveratrol from pruning waste of Vitis vinifera, fetească neagră variety. Appl. Sci. 13, 823–13. 10.3390/APP13020823

[B6] Gu L. Mo E. Yang Z. Zhu X. Fang Z. Sun B. (2007). Expression and localization of insulin-like growth factor-I in four parts of the red deer antler. Growth factors 25, 264–279. 10.1080/08977190701773187 18092234

[B7] Hassan S. A. (2005). Amino acid side chain interactions in the presence of salts. J. Phys. Chem. B 109, 21989–21996. 10.1021/JP054042R 16479276 PMC1366496

[B8] Huber V. Muller L. Hio J. Degot P. Touraud D. Kunz W. (2021). Improvement of the solubilization and extraction of curcumin in an edible ternary solvent mixture. Molecules 26, 7702–7726. 10.3390/MOLECULES26247702 34946787 PMC8703436

[B9] Karunnanithy V. Abdul Rahman N. H. B. Abdullah N. A. H. Fauzi M. B. Lokanathan Y. Min Hwei A. N. (2024). Effectiveness of lyoprotectants in protein stabilization during lyophilization. Pharmaceutics 16, 1346. 10.3390/pharmaceutics16101346 39458674 PMC11510631

[B10] Kasper J. C. Friess W. (2011). The freezing step in lyophilization: physico-chemical fundamentals, freezing methods and consequences on process performance and quality attributes of biopharmaceuticals. Eur. J. Pharm. Biopharm. 78, 248–263. 10.1016/J.EJPB.2011.03.010 21426937

[B11] Kawtikwar P. Bhagwat D. Sakarkar D. (2010). Deer antlers- Traditional use and future perspectives. Indian J. Traditional Knowl.

[B12] Khan M. Z. Zugaza J. L. Torres Aleman I. (2025). The signaling landscape of insulin-like growth factor 1. J. Biol. Chem. 301, 108047. 10.1016/J.JBC.2024.108047 39638246 PMC11748690

[B13] Kim C. R. Jeon H. L. Shin S. K. Kim H. J. Ahn C. W. Jung S. U. (2014). Neuroprotective action of deer bone extract against glutamate or Aβ_1-42_-induced oxidative stress in mouse hippocampal cells. J. Med. Food 17, 226–235. 10.1089/JMF.2013.2951 24460377

[B14] Kim H. S. Lim H. K. (1999). Inhibitory effects of velvet antler water extract on morphine-induced conditioned place preference and DA receptor supersensitivity in mice. J. Ethnopharmacol. 66, 25–31. 10.1016/S0378-8741(98)00195-0 10432204

[B15] Kim H. S. Lim H. K. Park W. K. (1999). Antinarcotic effects of the velvet antler water extract on morphine in mice. J. Ethnopharmacol. 66, 41–49. 10.1016/S0378-8741(98)00193-7 10432206

[B16] Kim K. H. Kim K. S. Choi B. J. Chung K. H. Chang Y. C. Lee S. D. (2005). Anti-bone resorption activity of deer antler aqua-acupunture, the pilose antler of Cervus Korean TEMMINCK var. mantchuricus Swinhoe (Nokyong) in adjuvant-induced arthritic rats. J. Ethnopharmacol. 96, 497–506. 10.1016/J.JEP.2004.09.039 15619570

[B17] Kim K. S. Choi Y. H. Kim K. H. Lee Y. C. Kim C. H. Moon S. H. (2004). Protective and anti-arthritic effects of deer antler aqua-acupuncture (DAA), inhibiting dihydroorotate dehydrogenase, on phosphate ions-mediated chondrocyte apoptosis and rat collagen-induced arthritis. Int. Immunopharmacol. 4, 963–973. 10.1016/J.INTIMP.2004.04.010 15182735

[B18] Kuo C. Y. Cheng Y. T. Ho S. T. Yu C. C. Chen M. J. (2018). Comparison of anti-inflammatory effect and protein profile between the water extracts from Formosan sambar deer and red deer. J. Food Drug Anal. 26, 1275–1282. 10.1016/J.JFDA.2018.02.005 30249326 PMC9298571

[B19] Li C. (2020). Deer antlers: traditional Chinese medicine use and recent pharmaceuticals. Anim. Prod. Sci. 60, 1233–1237. 10.1071/AN19168

[B20] Li C. An N. Song Q. Hu Y. Yin W. Wang Q. (2024). Enhancing organoid culture: harnessing the potential of decellularized extracellular matrix hydrogels for mimicking microenvironments. J. Biomed. Sci. 31, 96–25. 10.1186/S12929-024-01086-7 39334251 PMC11429032

[B21] Li C. Suttie J. M. Clark D. E. (2005). Histological examination of antler regeneration in red deer (*Cervus elaphus*). Anatomical Rec. - Part A Discov. Mol. Cell. Evol. Biol. 282, 163–174. 10.1002/ar.a.20148 15641024

[B22] Li L. Yang F. Jia R. Yan P. Ma L. (2020). Velvet antler polypeptide prevents the disruption of hepatic tight junctions via inhibiting oxidative stress in cholestatic mice and liver cell lines. Food Funct. 11, 9752–9763. 10.1039/D0FO01899F 33073799

[B23] Liu Y. Li H. Yang M. Guo J. Sun Z. Wang S. (2024). Sika deer velvet antler peptide exerts neuroprotective effect in a Parkinson’s disease model via regulating oxidative damage and gut microbiota. Pharm. (Basel) 17, 972. 10.3390/PH17070972 PMC11280472 39065820

[B24] Luo J. Huang J. Cong J. Wei W. Liu X. (2017). Double recognition and selective extraction of glycoprotein based on the molecular imprinted graphene oxide and boronate affinity. ACS Appl. Mater Interfaces 9, 7735–7744. 10.1021/ACSAMI.6B14733 28191926

[B25] Mahirogullari M. Ferguson C. M. Whitlock P. W. Stabile K. J. Poehling G. G. (2007). Freeze-dried allografts for anterior cruciate ligament reconstruction. Clin. Sports Med. 26, 625–637. 10.1016/J.CSM.2007.06.011 17920957

[B26] Martínez-Maqueda D. Hernández-Ledesma B. Amigo L. Miralles B. Gómez-Ruiz J. Á. (2013). Extraction/fractionation techniques for proteins and peptides and protein digestion. Proteomics Foods Princ. Appl., 21–50. 10.1007/978-1-4614-5626-1_2

[B27] Matejtschuk P. (2007). Lyophilization of proteins. Methods Mol. Biol. 368, 59–72. 10.1007/978-1-59745-362-2_4 18080462

[B28] Molnar A. Lakat T. Hosszu A. Szebeni B. Balogh A. Orfi L. (2021). Lyophilization and homogenization of biological samples improves reproducibility and reduces standard deviation in molecular biology techniques. Amino Acids 53, 917–928. 10.1007/S00726-021-02994-W 34002278 PMC8128086

[B29] Ortega Castillo N. Barallat Martínez De Osaba J. Alba Macias Y. Morales Indiano C. Doladé Botias M. Granada Ybern M. L. (2009). Estabilidad de las concentraciones de factor de crecimiento similar a la insulina tipo 1, gastrina y androstendiona en suero. Rev. del Lab. Clínico 2, 161–168. 10.1016/J.LABCLI.2009.06.001

[B30] Palma M. Barbero G. F. PiñEiro Z. Liazid A. Barroso C. G. Rostagno M. A. (2013). Extraction of natural products: principles and fundamental aspects. RSC Green Chem., 58–88. 10.1039/9781849737579-00058

[B31] Popova M. Bankova V. (2023). Contemporary methods for the extraction and isolation of natural products. BMC Chem. 17, 68–2. 10.1186/S13065-023-00960-Z 37391736 PMC10314546

[B32] Ren C. Gong W. Li F. Xie M. (2019). Pilose antler aqueous extract promotes the proliferation and osteogenic differentiation of bone marrow mesenchymal stem cells by stimulating the BMP-2/Smad1, 5/Runx2 signaling pathway. Chin. J. Nat. Med. 17, 756–767. 10.1016/S1875-5364(19)30092-5 31703756

[B33] Rossetti A. Chonco L. Alegría N. Zelli V. García A. J. Ramírez-Castillejo C. (2024). General direct anticancer effects of deer growing antler extract in several tumour cell lines, and immune system-mediated effects in xenograft glioblastoma. Pharmaceutics 16, 610. 10.3390/PHARMACEUTICS16050610 38794272 PMC11125008

[B34] Shi L. Zhao W. Yang Z. Subbiah V. Suleria H. A. R. (2022). Extraction and characterization of phenolic compounds and their potential antioxidant activities. Environ. Sci. Pollut. Res. 29, 81112–81129. 10.1007/S11356-022-23337-6 PMC9606084 36201076

[B35] Sun H. Xiao D. Liu W. Li X. Lin Z. Li Y. (2023). Well-known polypeptides of deer antler velvet with key actives: modern pharmacological advances. Naunyn Schmiedeb. Arch. Pharmacol. 397, 15–31. 10.1007/S00210-023-02642-Y 37555852

[B36] Tang Y. Fan M. Choi Y. J. Yu Y. Yao G. Deng Y. (2019). Sika deer (Cervus nippon) velvet antler extract attenuates prostate cancer in xenograft model. Biosci. Biotechnol. Biochem. 83, 348–356. 10.1080/09168451.2018.1537775 30381032

[B37] Tseng S. H. Chen L. G. Lai Y. J. Wang K. T. Wang C. C. (2016). Effects of different forages on the chemical compositions and antiosteoporotic activities of velvet antlers. Anim. Sci. J. 87, 989–996. 10.1111/ASJ.12536 26608104

[B38] Tseng S. H. Sung C. H. Chen L. G. Lai Y. J. Chang W. S. Sung H. C. (2014). Comparison of chemical compositions and osteoprotective effects of different sections of velvet antler. J. Ethnopharmacol. 151, 352–360. 10.1016/J.JEP.2013.10.060 24212078

[B39] Villaret-Cazadamont J. Poupin N. Tournadre A. Batut A. Gales L. Zalko D. (2020). An optimized dual extraction method for the simultaneous and accurate analysis of polar metabolites and lipids carried out on single biological samples. Metabolites 10, 338–10. 10.3390/METABO10090338 32825089 PMC7570216

[B40] Wang D. Landete-Castillejos T. (2023). Stem cells drive antler regeneration. Science 379, 757–758. 10.1126/SCIENCE.ADG9968 36821688

[B41] Wang J. Jia J. Song L. Gong X. Xu J. Yang M. (2018). Extraction, structure, and pharmacological activities of Astragalus polysaccharides. Appl. Sci. 2019, 122. 10.3390/APP9010122

[B42] Wu F. Li H. Jin L. Li X. Ma Y. You J. (2013). Deer antler base as a traditional Chinese medicine: a review of its traditional uses, chemistry and pharmacology. J. Ethnopharmacol. 145, 403–415. 10.1016/J.JEP.2012.12.008 23246455

[B43] Xia P. Liu D. Jiao Y. Wang Z. Chen X. Zheng S. (2022). Health effects of peptides extracted from deer antler. Nutrients 14, 4183. 10.3390/NU14194183 36235835 PMC9572057

[B44] Yang H. Wang L. Sun H. He X. Zhang J. Liu F. (2017). Anticancer activity *in vitro* and biological safety evaluation *in vivo* of Sika deer antler protein. J. Food Biochem. 41, e12421. 10.1111/JFBC.12421

[B45] Yang Z. Li Y. Xue W. Yin Z. Meng Z. Zhou A. (2021). Small molecules from multistep extraction of coal and their effects on coal adsorption of CH4. Catal. Today 374, 192–199. 10.1016/J.CATTOD.2020.09.009

[B46] Yao B. Zhang M. Leng X. Liu M. Liu Y. Hu Y. (2018). Antler extracts stimulate chondrocyte proliferation and possess potent anti-oxidative, anti-inflammatory, and immune-modulatory properties. Vitro Cell Dev. Biol. Anim. 54, 439–448. 10.1007/S11626-018-0266-2 29850973

[B47] Yao B. Zhang M. Leng X. Zhao D. (2019). Proteomic analysis of the effects of antler extract on chondrocyte proliferation, differentiation and apoptosis. Mol. Biol. Rep. 46, 1635–1648. 10.1007/S11033-019-04612-1 30680597

[B48] Zhang G. Shi L. Li J. Wang S. Ren J. Wang D. (2023a). Antler stem cell exosomes alleviate pulmonary fibrosis via inhibiting recruitment of monocyte macrophage, rather than polarization of M2 macrophages in mice. Cell Death Discov. 9, 359–12. 10.1038/S41420-023-01659-9 37770458 PMC10539297

[B49] Zhang G. Wang D. Ren J. Li J. Guo Q. Shi L. (2023b). Antler stem cell-derived exosomes promote regenerative wound healing via fibroblast-to-myofibroblast transition inhibition. J. Biol. Eng. 17, 67–14. 10.1186/S13036-023-00386-0 37940994 PMC10633995

[B50] Zhang L. Z. Xin J.Le Zhang X. P. Fu Q. Zhang Y. Zhou Q. L. (2013). The anti-osteoporotic effect of velvet antler polypeptides from *Cervus elaphus* Linnaeus in ovariectomized rats. J. Ethnopharmacol. 150, 181–186. 10.1016/J.JEP.2013.08.029 23993908

[B51] Zhang Q. W. Lin L. G. Ye W. C. (2018). Techniques for extraction and isolation of natural products: a comprehensive review. Chin. Med. (United Kingdom) 13, 20–26. 10.1186/S13020-018-0177-X PMC5905184 29692864

[B52] Zhao L. Wang X. Zhang X. L. Xie Q. F. (2016). Purification and identification of anti-inflammatory peptides derived from simulated gastrointestinal digests of velvet antler protein (*Cervus elaphus* Linnaeus). J. Food Drug Anal. 24, 376–384. 10.1016/J.JFDA.2015.10.003 28911592 PMC9339547

[B53] Zheng K. Li Q. Lin D. Zong X. Luo X. Yang M. (2020). Peptidomic analysis of pilose antler and its inhibitory effect on triple-negative breast cancer at multiple sites. Food Funct. 11, 7481–7494. 10.1039/D0FO01531H 32789330

[B54] Zhou J. Zhao J. Wang Y. Jiang Y. Li X. Wang D. (2024). Repair of mechanical cartilage damage using exosomes derived from deer antler stem cells. Front. Biosci. 29, 309. 10.31083/J.FBL2908309 39206920

